# Visual policy narrative messaging improves COVID-19 vaccine uptake

**DOI:** 10.1093/pnasnexus/pgad080

**Published:** 2023-04-18

**Authors:** Elizabeth A Shanahan, Rob A DeLeo, Elizabeth A Albright, Meng Li, Elizabeth A Koebele, Kristin Taylor, Deserai Anderson, Katherine L Dickinson, Honey Minkowitz, Thomas A Birkland, Manli Zhang

**Affiliations:** Department of Political Science, Montana State University, Bozeman, MT 59717, USA; Department of Global Studies, Bentley University, Waltham, MA 02452, USA; Division of Environmental Science and Policy, Nicholas School of the Environment, Duke University, Durham, NC 27708, USA; School of Public Affairs, University of Colorado, Denver, CO 80217, USA; Department of Political Science, University of Nevada, Reno, NV 89557, USA; Department of Political Science, Wayne State University, Detroit, MI 48202, USA; School of Public Affairs, University of Colorado, Denver, CO 80217, USA; School of Public Affairs, University of Colorado, Denver, CO 80217, USA; Department of Public Administration, School of Public and International Affairs, North Carolina State University, Raleigh, NC 27695, USA; Department of Public Administration, School of Public and International Affairs, North Carolina State University, Raleigh, NC 27695, USA; School of Public Affairs, University of Colorado, Denver, CO 80217, USA

**Keywords:** narrative risk communication, visual policy narratives, COVID-19, vaccine behavior, risk perception

## Abstract

In the face of vaccine hesitancy, public health officials are seeking more effective risk communication approaches to increase vaccination rates. We test the influence of visual policy narratives on COVID-19 vaccination behavior through a panel survey experiment conducted in early 2021 (*n* = 3,900) and then 8 weeks later (*n* = 2,268). We examine the effects of three visual policy narrative messages that test the narrative mechanism of character selection (*yourself*, *your circle*, and *your community*) and a nonnarrative control on COVID-19 vaccine behavior. Visual risk messages that use narratives positively influence COVID-19 vaccination through serial mediation of affective response to the messages and motivation to get the COVID-19 vaccination. Additionally, character selection matters, as messages focusing on protecting others (i.e. your *circle* and *your community*) perform stronger than those of *yourself*. Political ideology moderated some of the effects, with conservative respondents in the nonnarrative control condition having a higher probability of vaccination in comparison to the *protect yourself* condition. Taken together, these results suggest that public health officials should use narrative-based visual communication messages that emphasize communal benefits of vaccinations.

**Significance Statement**

To increase individuals’ risk reduction behaviors in times of crisis, how risk messages are communicated matters. In this experimental panel study on COVID-19 vaccine behavior, we find three key effective risk communication strategies. First, relaying information in narrative form, which includes characters and a moral to the story (solution), is more powerful at influencing vaccine behavior than nonnarrative, take action–only, messages. Second, narrative messages focusing on protecting others familiar to you as opposed to oneself are most effective across all political ideologies. Third, visual policy narrative messages can be a productive approach for risk communication across various venues, such as social media, for effective risk communication and to effectively foster public health.

## Introduction

Vaccines have been exceptionally successful in the eradication of debilitating and life-threatening infectious diseases such as smallpox and polio (1). However, vaccine hesitancy in the United States and other countries has remained constant over time (2), despite increasingly sophisticated and well-funded technologies for the development and efficacy testing of vaccines. This dynamic has been clearly on display during the COVID-19 pandemic. To date (December 2022), only 68.6% of Americans are fully COVID-19 vaccinated, with rates in some states as low as 52.6% (3). With an eye toward diminishing viral spread and achieving herd immunity, the health community is calling for more effective risk communication approaches to raise awareness, align attitudes, and positively influence vaccine uptake. In tandem with critical works on crisis and risk communication (4), we propose to explore a narrative-based risk communication approach (5), whereby risk information is conveyed in story form as a *lingua franca* to influence risk reduction behavior (6).

**Fig. 1.**
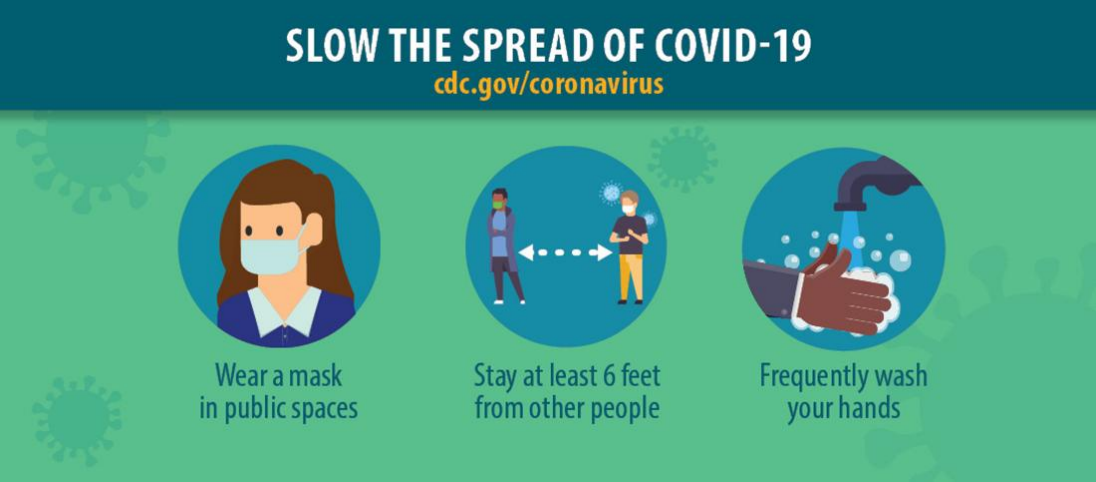
Example of early nonnarrative communication from CDC that employs imperative statements and visual graphics. Found at cdc.gov/coronavirus in 2000.

The goal of effectively communicating technical scientific information about risks and benefits of hazard preparation and mitigation is to improve people's risk preparedness and reduction decisions (7). Since the advent of COVID-19 vaccines, entities such as the Center for Disease Control and the U.S. Department of Health and Human Services have worked to improve vaccine communications (8, 9), in part, but perhaps inadvertently, through the use of narrative structure. For example, “COVID-19 Vaccination is the best way to protect children against severe COVID-19” (10) is a narrative message that includes a victim (children) who could be harmed by a villain (COVID-19) but can be protected by a hero who provides them with a solution (the COVID-19 vaccine). However, there is also an array of nonnarrative messages that take the form of imperative statements, such as “Get Ready for School with COVID-19 Vaccinations” (10), that do not contain characters. Indeed, in the early months of COVID-19, much of the risk communication came in the form of such nonnarrative imperative statements (Fig. 1). While researchers find that narratives influence risk perceptions and behavior more effectively than nonnarrative messages across topical domains (e.g. health (11, 12), disasters (6)), what is neither clearly identified nor understood are the specific *narrative mechanisms* that lead to greater persuasion.

The power of narratives is rooted in the central nature of stories in our lives, which both shape and reflect our values, beliefs, and identity. As *homo narrans* (13), we interpret information and make meaning of the world around us through narratives. The theoretical scaffolding for examining the relationship between narrativity and behavior in the context of public policies can be found in the Narrative Policy Framework (NPF) (14), which asserts that narratives have universal structural elements that can be systematically measured across different contexts and thus generalized to improve risk communication efforts. The NPF contends that a “policy narrative” must include at least one character (e.g. hero, villain, and victim) and a moral to the story that refers to a solution or call to action (15). These narrative elements, among others such as plot and setting, serve as narrative mechanisms to persuade the target audience.

While extant research has discovered the power of narrative persuasion at a coarse level (i.e. narrative versus nonnarrative form), this study advances the science of narrative risk communication by testing the effects of a specific narrative mechanism, character selection (16), on outcome behavior within the context of COVID-19 vaccinations. Additionally, since most NPF studies focus on textual policy narratives, this study also leverages visual or graphic messaging, which aligns with contemporary public health approaches (e.g. Fig. 1). We thus test the effects of “visual policy narratives” or narratives embedded within and communicated through graphic stimuli.

In the context of the COVID-19 pandemic, emerging research has found that persuasive messages about vaccinations are effective when they emphasize personal health risks and collective health outcomes, regardless of who sends the message (17). The implications for vaccine uptake are complicated by findings suggesting that (i) individual perceptions of vaccine attributes influence vaccine uptake (18) and (ii) partisan leaning influences vaccine uptake choices, with higher vaccination rates with Democrats than Republicans (19). Although these findings suggest that individuals are influenced by messages, that influence is conditional on the characteristics of the vaccine and individual political preferences. In this study, we seek to understand the influence of the message structure itself, notably via a visual platform of risk messaging.

While risk messages have been typically conveyed as verbal (speeches) or written (newsletters, media accounts, and agency statements) communications, in the age of social media and other online communication, many risk messages are now designed to be brief and include visual stimuli to influence individual behavior and attitudes (20). Indeed, in simulated Facebook postings, narrative text paired with an image has been found to amplify an individual's affective response and policy support, in contrast to narrative text alone (21). Our analysis of visual policy narrative messages thus accounts for the need for risk communication to be further adapted to social media and other visually based platforms by embedding narrative words in graphic imagery to test effects on behavior. As public officials increasingly rely on various visual platforms for risk, health, and safety marketing campaigns to shape individual behavior (22), understanding the persuasive power of these visual policy narrative messages is essential to advance the science of risk communication.

Risk communication does not occur in a vacuum, however. Indeed, the efficacy of any risk message is also determined by a host of related variables that mediate and/or moderate the influence of risk messages on outcome behaviors:

- An individual's *affective response* to a risk message is increasingly found to be a mediating psychological experience that influences behavioral choices (14).
- One's *intention* or motivation to act is sometimes a mediating psychological process that influences behavior choices (23).
- *Beliefs* shape how individuals perceive and make decisions about risk (24). Particularly within highly politicized contexts including the current political and social climate associated with the COVID-19 pandemic and the vaccine outreach campaign more specifically, political ideology can serve as an important proxy for individual beliefs (25).
- *Personal characteristics*, such as gender, age, race, and socioeconomic status (26), may also influence how an individual perceives risks, thereby impacting their receptivity to different risk messages.
- *Risk perception* may be a mediating psychological experience that influences behavioral choices. Risk perception is defined by two dimensions: the perceived probability or likelihood of a risk occurring and the perceived consequences or severity of that occurrence (27). Indicators of risk perception commonly include measures of probability, worry, and fear about a risk and its implications (28).

**Fig. 2.**
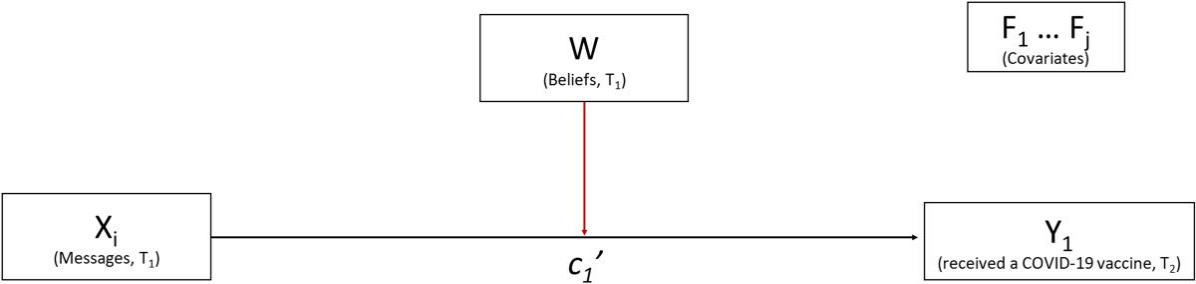
*T*_1_, wave 1; *T*_2_, wave 2; *X_i_*, experimental conditions; *Y*_1_, outcome variable (received a COVID-19 vaccine); *W*, moderating variable (beliefs); *F*_1…*j*_, covariates (risk perception, COVID-19 experience, flu vaccine history, and demographics).

**Fig. 3.**
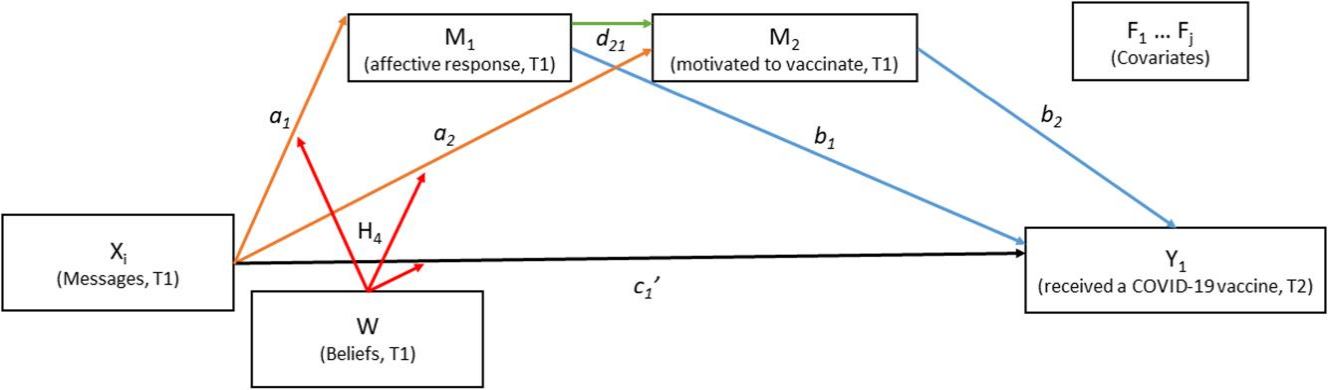
*T*_1_, wave 1; *T*_2_, wave 2; *X_i_*, experimental conditions; *M*_1–2_, mediators (affective response and motivated to vaccinate); *Y*_1_, outcome variable (received a COVID-19 vaccine); *W*, moderating variable (beliefs); *F*_1…*j*_, covariates (risk perception, COVID-19 experience, flu vaccine history, and demographics).

The aim of this study is to advance the science of narrative risk communication about COVID-19 vaccination through five objectives:

1. Assess the effects of visual policy narrative messages and specific narrative mechanisms (character selection) on vaccine behavior (Fig. 2).
2. Assess the moderation effects of political ideology on vaccine behavior (Fig. 2).
3. Assess the mediation effects of affective response and motivation to vaccinate on vaccine behavior (Fig. 3).
4. Assess the moderation effects of political ideology on mediation effects of affective response and motivation to vaccinate on vaccine behavior (Fig. 3).
5. Assess the effects of covariates (risk perception, COVID-19 experience, vaccine history, and demographics) on mediator, moderator, and outcome variables (Fig. 3).

**Fig. 4.**
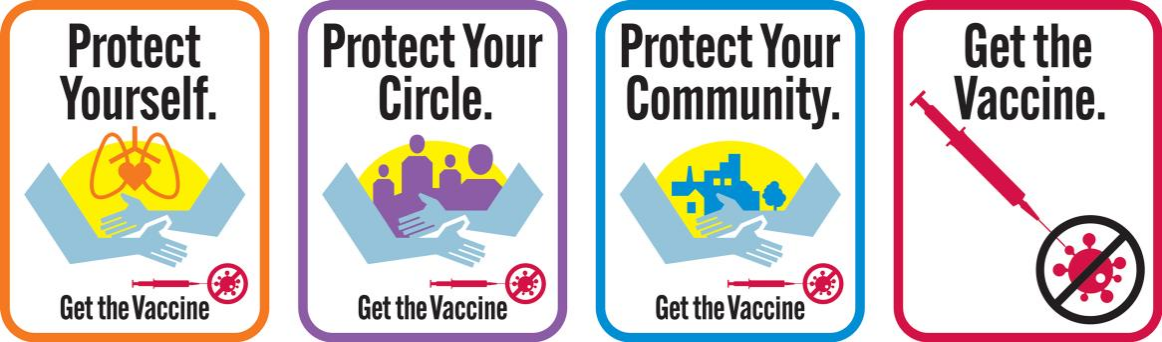
Three treatment conditions: protect yourself, protect your circle, and protect your community; control condition: get the vaccine. Graphics produced by Todd Radom.

To accomplish these objectives, we conducted a panel survey experiment, which was distributed to a national sample of US citizens (*n* = 3,900) in 2021. Of four experimental conditions (*X_i_* in Fig. 3), three were treatment conditions (visual policy narrative messages) and one was a control (visual nonnarrative message) (Fig. 4). Because a foundational feature of what constitutes a narrative is the presence of characters (15), this study tests the narrative mechanism of character *selection* in the treatment conditions. Each visual policy narrative message contains a different “victim” character, or those most at risk from COVID-19 and range from proximal to distal entities (29): “yourself,” “your circle,” or “your community.” The “hero” character (the individual taking the survey who “protects” a victim) and the moral or solution (get the vaccine) are held constant across all conditions. The control condition features only the moral and contains no characters, making it nonnarrative. Respondents were randomly assigned to one of the four experimental conditions in *T*_1_; the outcome variable, COVID-19 vaccine behavior, was collected 8 weeks later in *T*_2_. We used Hayes PROCESS macro (30), a regression-based method, to analyze total, mediation, and moderation effects of visual policy narratives on vaccination behavior.

## Results

### Analysis 1. Effect of message conditions on vaccination, moderated by political ideology

We conducted a logistic regression to measure the effects of experimental message conditions, political ideology, and their interaction on predicting COVID-19 vaccination status, controlling for covariates. We coded the multicategorical message conditions in two ways (30), resulting in two versions of this regression (Table 1): indicator coding (dummy coding) that compares each treatment condition to the control condition (p. 68) and Helmert coding (p. 226), which is a series of orthogonal contrasts that compares each experimental condition to the average of the subsequent conditions (average of three narrative messages vs. control; the average of “circle” and “community” messages vs. the “yourself” message; the “community” message vs. the “circle” message). The former reveals the impacts of each visual policy narrative condition, and the latter enables a comparison between means of assemblages of visual narrative conditions. This analysis explores Objectives 1 and 2.

**Table 1.** Total effects on vaccine behavior for indicator and Helmert coding.

	Indicator coding *b* (se) with covariates	Indicator coding *b* (se) without covariates	Helmert coding *b* (se) with covariates	Helmert coding *b* (se) without covariates
Constant	−4.742*** (0.3440)	−0.2601** (0.0853)	−4.751*** (0.3324)	−0.1717 (1176)
*X*_1_	−0.2270 (0.1597)	−0.1048 (0.1203)		
*X*_2_	.2288 (0.1584)	.1717 (0.1203)		
*X*_3_	−0.0367 (0.1587)	−0.0250 (0.1208)		
Political ideology	−0.0474 (0.0722)	−0.0949 (0.0550)		
*X*_1_ * political ideology	.2207* (0.1009)	.1867* (0.0769)		
*X*_2_ * political ideology	.0361 (0.1007)	.0049 (0.0771)		
*X*_3_ * political ideology	.0551 (0.0984)	.111 (0.0763)		
*X*_4_			−0.0116 (0.1306)	−0.3961 (0.2730)
*X*_5_			.3230* (0.1368)	.7012* (0.2910)
*X*_6_			−0.2655 (0.1560)	−0.6282 (0.3272)
Political ideology			.0306 (0.0722)	
*X*_4_ * political ideology			.1040 (0.0822)	
*X*_5_ * political ideology			−0.1751* (0.0863)	
*X*_6_ * political ideology			.0191 (0.0976)	
Risk perception—severity	.0299* (0.0149)		.0299* (0.0149)	
Risk perception—likelihood	.0197 (0.0174)		.0197 (0.0174)	
COVID experience	.1848 (0.1619)		.1848 (0.1619)	
Flu shot history	.5097*** (0.0465)		.5097*** (0.0465)	
Race	.4472** (0.1493)		.4472** (0.1493)	
Gender	−0.0291 (0.1204)		−0.0291 (0.1204)	
Age	.0453*** (0.0041)		.0453*** (0.0041)	
Education	.1647*** (0.0436)		.1647*** (0.0436)	
Income	.0870*** (0.0238)		.0870*** (0.0238)	
Children	−0.2794*** (0.0644)		−0.2794*** (0.0644)	

*P < 0.05; **P < 0.01; ***P < 0.001. Bold indicates statistical significance.

*X*_1_, protect yourself compared to control; *X*_2_, protect circle compared to control; *X*_3_, protect community compared to control; *X*_4_, narrative (average of protect yourself, protect your circle, and protect your community) compared to control; *X*_5_, average of protect circle and protect your community compared to protect yourself; *X*_6_, protect community compared to protect your circle.

Level of confidence for all confidence intervals = 95. Number of bootstrap samples for percentile bootstrap confidence intervals: 5000. Political ideology was mean centered. Hayes (2022) Model 1.

First, we examine the extent to which exposure to a treatment condition in *T*_1_ affects behavior in *T*_2_ (Obj 1). In the logistic regression with indicator coding, we find no total effect of any of the visual policy narrative treatments on vaccination behavior, controlling for political ideology and covariates. However, the Helmert coding shows that message conditions focusing on protecting others (the mean of “protect your circle” and “protect your community”) lead to higher odds of COVID-19 vaccination in comparison with the more proximate “protect yourself” (*b* = 0.3230, *P* = 0.02). Additionally, the “protect your circle” condition leads to marginally higher vaccination rate than the “protect your community” conditions (*b* = −0.2655, *P* = 0.09). The average of all three narrative message conditions does not differ from the control condition (*b* = −0.0116, *P* = 0.93). These results suggest that a focus on protecting “victim” groups outside of oneself leads to a greater probability of COVID-19 vaccine uptake, in comparison to protecting oneself. Moreover, the message focusing on protecting one's close circle appears to be the most effective way to motivate COVID-19 vaccination. In sum, the victim character selection is a narrative mechanism that influences risk reduction behavior.

**Fig. 5.**
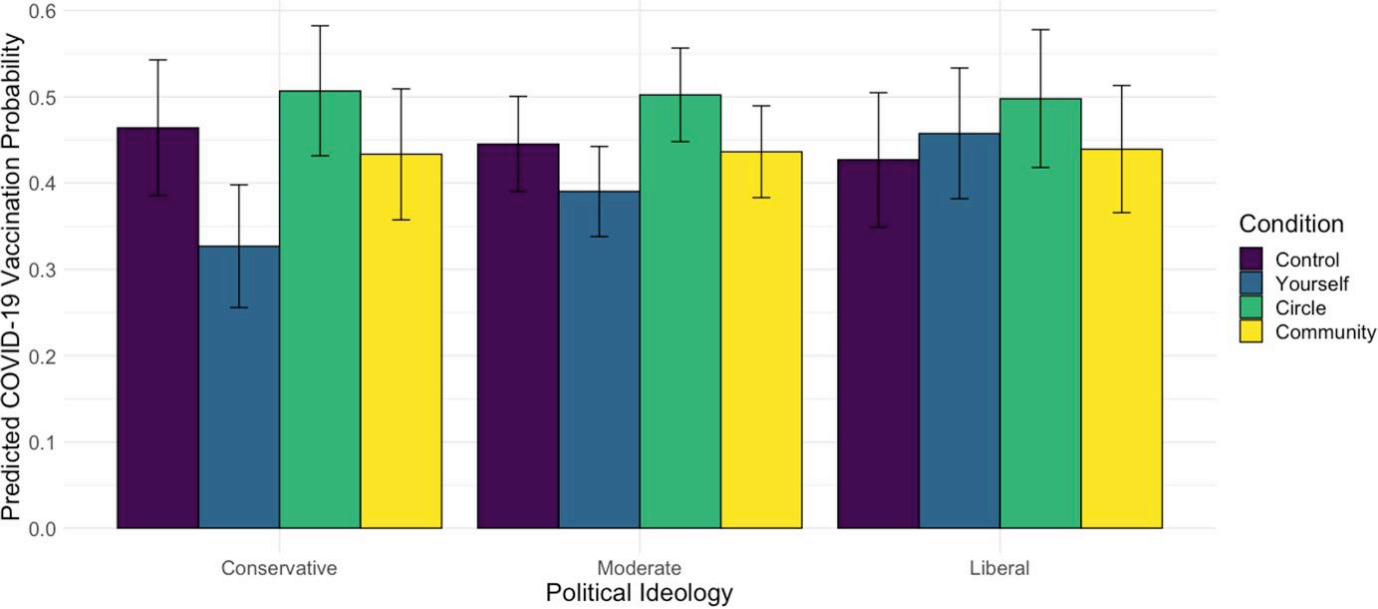
Predicted probability of COVID-19 vaccination at *T*_2_, by messaging condition and political ideology at *T*_1_, at mean levels of perceptions of severity of risk, likelihood of risk, age, education, income, COVID-19 infection experience, flu vaccine history, gender, race (white vs. minority), and number of children. Predicted probability of moderates were calculated on the mean of political ideology; liberals at +1 SD and conservatives at −1 SD. Error bars were calculated at the 95% confidence level with standard errors estimated with the delta method using Stata.

Next, we assess if political ideology, a proxy for beliefs in this politicized context, moderates the effect of a message condition on vaccination behavior (Obj 2). As the indicator coding reveals, there is a significant interaction between the “protect yourself” condition and political ideology (*b* = 0.2207, *P* = .03), where the treatment effect of “protect yourself” message is less than that of the control condition for more conservative participants. Indeed, the predicted probability of COVID-19 vaccination by experimental condition and political ideology (Fig. 5) reveals conservative respondents in the control condition had a 46% probability of being vaccinated at *T*_2_, while those in the “protect yourself” condition had a 33% probability of vaccination, revealing a difference of 13 percentage points and holding covariates constant. When exposed to the “protect your circle” and “protect your community” conditions, conservative respondents have a 51% and 43% probability of being vaccinated at *T*_2_, respectively. Helmert coding shows that political ideology also has a significant interaction, whereby the treatment effect for protecting others (mean of “protect your circle” and “protect your community”) has a stronger effect than the individual focused “protect yourself” message on vaccine uptake in *T*_2_ among conservatives (*b* = −0.1751, *P* = 0.04).

The overall pattern is similar for political moderates, though the difference in vaccination probability between the control (45%) and the “protect yourself” (39%) reveals a 6-percentage point difference, smaller than among conservatives. Vaccination probabilities among moderates exposed to the “protect your circle” (50%) and “protect your community” (44%) are equivalent to conservatives. Interestingly, predicted vaccination probabilities in the control condition are lower among liberals (43%) compared to other groups, and all three treatment conditions reveal higher vaccination rates in this group (46% in the “protect yourself” condition, 50% in the “protect your circle” condition, and 44% in the “protect your community” condition). These effects are controlling for other differences across these groups (e.g. age, race/ethnicity, and education) given that these effect estimates are computed holding other covariates at means or reference levels.

Analysis on the simple slopes of political ideology for each message condition shows that participants with different political ideologies did not differ on vaccination rate following exposure to the control message (*b* = −0.047, *P* = 0.51), the “protect your circle” message (*b* = −0.011, *P* = 0.84), or the “protect your community” message (*b* = −0.008, *P* = 0.91). Yet, political ideology did affect COVID-19 vaccination behavior in the “protect yourself” message condition: more conservative participants demonstrated a significantly lower likelihood to vaccinate than more liberal participants in this condition (*b* = −0.173, *P* = 0.01).

### Analysis 2. Moderated mediation for the effect of message conditions on vaccination

To explore Objectives 3, 4, and 5, we conducted a moderated mediation analysis that added two critical variables to the model: affective response and motivation to vaccinate (Fig. 2). Specifically, (i) the independent variable is the message condition (*X_i_*) at *T*_1_, coded using indicator contrast, (ii) the outcome variable is COVID-19 vaccination behavior (*Y*_1_) at *T*_2_, (iii) the serial mediators are affective response (*M*_1_ at *T*_1_) and motivation to vaccinate (*M*_2_ at *T*_1_), (iv) the moderating variable is political ideology (W at *T*_1_), moderating the a_1_, a_2_, and c_1_' paths, and (v) the covariates are risk perception, COVID-19 experience, history of receiving the flu vaccine, and demographics.

First, we explore the extent to which respondents’ affective responses (*M*_1_) to the risk message (*X_i_*) and ensuing motivation to get the COVID-19 vaccine (*M*_2_) are mediating psychological experiences for the effect of narrative messages on vaccination behavior (Obj 3). We find that visual policy narrative messages do influence vaccine behavior through a process of serial mediation. Compared to the control condition, each of the three visual policy narrative treatments has a significant positive indirect effect on vaccine behavior through the serial mediation pathway *message condition → affective response → motivation to vaccination → COVID-19 vaccination*, (Table 2 and Fig. 2), with the indirect effects for “yourself” (0.0872, 95% CI = 0.0433, 0.1425), “your circle” (0.0941, 95% CI = 0.0481, 0.1508), and “your community” (0.1022, 95% CI = 0.0518, 0.1641) conditions (Table 3). In sum, each of the three visual policy narrative conditions leads to more positive affective responses, which in turn leads to higher motivation to get the COVID-19 vaccine, which in turn leads to more vaccination behavior.

**Table 2.** Moderated mediation models with interactions and covariates.

Variable	Model 1 Affective response (*M*_1_;*T*_1_) *b* (se)	Model 2 Motivated to vaccinate (*M*_2_;*T*_1_) *b* (se)	Model 3 Got COVID-19 vaccine (*Y*_1_;*T*_2_) *b* (se)
*X*_1_ (Fig. 2; pathway ***a*_1_**)	.6288*** (0.0963)	−0.2463* (0.1007)	−0.2809 (0.1695)
*X*_2_ (Fig. 2; pathway ***a*_1_**)	.6785*** (0.0970)	−0.1202 (0.1016)	.1010 (0.1691)
*X*_3_ (Fig. 2; pathway ***a*_1_**)	.7370*** (0.0966)	−0.1512 (1015)	−0.1045 (0.1683)
Affective response (*M*_1_) (Fig. 2; pathway ***d*_21_**)		.8154*** (0.0241)	.0145 (0.0523)
Motivated to vaccinate (*M*_2_) (Fig. 2; pathway ***b*_2_**)			.1701*** (0.0390)
*X*_1_ * political ideology	−0.1130 (0.0615)	−0.0078 (0.0637)	.2124** (0.1066)
*X*_2_ * political ideology	−0.0169 (0.0622)	−0.0084 (0.0643)	−0.0388 (0.1075)
*X*_3_ * political ideology	−0.0424 (0.0607)	.0415 (0.0628)	.0488 (0.1038)
Risk perception: likelihood	.0049 (0.0110)	.0311** (0.0114)	.0026 (0.0187)
Risk perception: severity	.0575*** (0.0089)	.0180 (0.0093)	.0243 (0.0158)
COVID-19 experience	.1190 (0.1021)	−0.1744 (0.1057)	.1544 (0.1726)
Flu vaccine frequency	.2193*** (0.0293)	.1925*** (0.0308)	.4320*** (0.0493)
Race	−0.1862* (0.0928)	−0.1149 (0.0960)	.4140** (0.1566)
Gender	−0.1876* (0.0742)	−0.2059** (0.0768)	.0195 (0.1270)
Age	.0119*** (0.0025)	.0069** (0.0026)	.0442*** (0.0043)
Education	.0289 (0.0266)	.0120 (0.0275)	.1580*** (0.0456)
Income	.0217 (0.0145)	−0.0100 (0.0150)	.0736** (0.0248)
Kids	−0.0540 (0.0366)	.0511 (0.0379)	−0.3167*** (0.0696)
Political ideology	.1272** (0.0443)	−0.0110 (0.0460)	−0.0382 (0.0764)

* *P* < 0.05; ** *P* < 0.01; *** *P* < 0.001. Bold indicated statistical significance.

*X*_1_, protect yourself compared to control; *X*_2_, protect circle compared to control; *X*_3_, protect community compared to control.

Hayes (2022) Model 85.

**Table 3.** Indirect effects in serial mediation.

Message → affective response → motivated to vaccinate → COVID-19 vaccination behavior				
Indicator	Effect	BootSE	BootLLCI	BootULCI
*X*_1_	.0872	.0254	.0433	.1425
*X*_2_	.0941	.0262	.0481	.1508
*X*_3_	.1022	.0283	.0518	.1641

* *P* < 0.05; ** *P* < 0.01; *** *P* < 0.001.

*X*_1_, protect yourself compared to control; *X*_2_, protect circle compared to control; *X*_3_, protect community compared to control.

Level of confidence for all confidence intervals = 95. Number of bootstrap samples for percentile bootstrap confidence intervals: 5000. Hayes (2022) Model 85.

In contrast, the pathways that tested affective response (pathways *a_1_* and *b_1_*; Fig. 2) and motivation to vaccinate (pathways *a_2_* and *b_2_*; Fig. 2) as single mediators did not emerge as significant indirect effects with one exception (Supplement A). In comparison to the control condition, the effect of the “protect yourself” message has a negative mediated effect on vaccination through motivation, (−0.0419, 95% CI = −0.0871, −0.0071), which is driven by the negative effect of the “protect yourself” message on motivation to vaccinate, (Table 2, Model 2; *b* = −0.2463, *P* = 0.0146).

To evaluate the moderating effects of political ideology on the mediated pathways, we examine the index of moderated mediation, which quantifies the linear association between the indirect effect of mediators and the moderation of that effect by political ideology (Obj 4). The index of moderated mediation reveals no significant moderated mediation by political ideology, indicating that the mediated effect of message condition on COVID-19 vaccination behavior through affective response and motivation to vaccinate did not vary by political ideology (Supplement A). However, political ideology did serve as a significant moderator for the direct effect of the “protect yourself” condition (vs. control) in the model (Table 2, Model 3; *b* = 0.2124, *P* = 0.0464).

Finally, we assess the effects of covariates (risk perceptions, COVID-19 experience, vaccine history, and demographics) on mediator, moderator, and outcome variables (Obj 5). We assessed the sensitivity of the main effects model to covariates and found no change to the results of the model without the covariates (Supplement B). Risk perception is operationalized in two dimensions: the perceived likelihood of effects of getting COVID-19 and the severity of the disease's effects (Table 4). Respondents who are more worried about the impact of COVID-19 (higher perceived severity) had a more positive affective response to the risk messages (Table 2, Model 1; *b* = 0.0575, *P* < 0.001). Respondents who perceived that they were more likely to get COVID-19 were more motivated to get the vaccine (Table 2, Model 2; *b* = 0.0311, *P* = 0.0062). In Model 3 (Table 2), neither perceived likelihood of getting COVID-19 (*b* = 0.0026, *P* = 0.8886) nor the perceived severity of the disease (*b* = 0.0243, *P* = 0.1250) was a significant predictor for vaccination. However, in the regression for vaccination that did not include mediators as predictors (Table 1), perceived severity was a significant predictor of vaccination (*b* = 0.0299, *P* = 0.0455). Thus, the two dimensions of risk perception do not operate in a monolithic fashion.

**Table 4.** Variable summary.

Name	Description	Values	Descriptives
*Dependent, moderating, and mediating variables*			
*Vaccine behavior* (*Y*_1_; *T*_2_)	Received one or more COVID-19 vaccine doses	0 = no; 1 = yes	$\bar{X}$ = 0.44 *n* = 2268
*Affective response* (*M*_1_; *T*_1_)	Rate reaction to message	1 = extremely negative to 7 = extremely positive	$\bar{X}\;$ = 4.84 s.d. = 1.614 *n* = 3884
*Motivation to vaccinate* (*M*_2_; *T*_1_)	Motivate to get COVID-19 vaccine after image exposure	1 = not at all to 7 = a great deal	$\bar{X}\;$ = 3.79 s.d. = 2.067 *n* = 3493
*Political ideology* (*W*; *T*_1_)	Political ideology scale	1 = extremely conservative to 7 = extremely liberal	$\bar{X}$ = 4 s.d. = 1.598 *n* = 3900
*Covariates* (all *T*_1_)			
*Risk perception: likelihood*	Multi-item: likelihood to (i) get COVID-19, (ii) get seriously ill, and (iii) die	1 = not a chance to 19 = certain Cronbach's alpha = 0.805	$\bar{X}$ = 7.42 s.d. = 3.721 *n* = 3885
*Risk perception: severity*	Multi-item: worry about COVID-19 (i) getting it, (ii) others I care about getting it, (iii) feel anxious, and (iv) feel uncomfortable	1 = strongly disagree to 17 = strongly agree Cronbach's alpha = 0.864	$\bar{X}$ = 10.84 s.d. = 4.347 *n* = 3900
*COVID-19 experience*	Had COVID-19	0 = no; 1 = yes	$\bar{X}$ = 0.15 *n* = 3517
*Flu vaccine frequency*	How often get flu vaccine	1 = never to 4 = every year	$\bar{X}$ = 1.59 s.d. = 1.24 *n* = 3900
*Race*	All other races—White	0 = all other races 1 = White	$\bar{X}$ = 0.24 *n* = 3900
*Gender*	Male—female/nonbinary	0 = male 1 = female/nonbinary	$\bar{X}$ = 0.50 *n* = 3887
*Age*	Age in years	18 to 93	$\bar{X}$ = 45.05 s.d. = 17.594 *n* = 3900
*Education*	Levels of education	1 = <high school to 6 = graduate degree	$\bar{X}$ = 3.94 s.d. = 1.487 *n* = 3900
*Income*	Household pretax income in 2020	1 = $10k or less to 10 = >$150k	$\bar{X}$ = 5.81 s.d. = 2.817 *n* = 3900
*Children*	Number of children < 18 years	0 to 10	$\bar{X}$ = 0.67 s.d. = 1.017 *n* = 3889

Two covariates have a significant impact across all three models (Table 2): respondents’ history of receiving the flu vaccine and age. Respondents who reported getting the flu vaccine more consistently in the past had a higher affective response (*b* = 0.2193, *P* < 0.001), motivation to vaccinate (*b* = 0.1925, *P* < 0.001), and were more likely to receive at least one dose of the COVID-19 vaccine (*b* = 0.4320, *P* < 0.001). Conversely, respondents’ experience with COVID-19 did not have a significant impact on any of the outcome variables. Older respondents had a higher affective response (*b* = 0.0119, *P* < 0.001) and motivation to vaccinate (*b* = 0.0069, *P* = 0.0074), and they were more likely to receive at least one dose of the COVID-19 vaccine (*b* = 0.0442, *P* < 0.001). Respondents with more education (*b* = 0.1580, *P* = 0.0005), higher income (*b* = 0.0736, *P* = 0.0030), and fewer children (*b* = −0.3167, *P* < 0.001) were all more likely to receive at least one dose of the COVID-19 vaccine, with no impact on affective response or motivation to vaccinate.

## Discussion

The public health community is urgently calling (31) for more effective communication approaches to encourage COVID-19 vaccination in order to achieve herd immunity, lower stress on hospitals, and ultimately save lives. While extant research has identified risk communication techniques that may alter behavior (32), significant challenges remain for realizing concomitant risk reduction across multiple hazard domains (33). Using narrative-based messages to enhance risk communication (6) can help close this efficacy gap.

Communicating science and/or suggested behaviors in narrative form has been found to be more effective than information-only risk messages (11). This study advances the science of narrative risk communication by analyzing the effects of specific narrative mechanisms, situated within visual policy narrative messages, on vaccination behavior. Further, we leverage the NPF (14) as a theoretical anchor to bring precision and replicability to research on narrative risk communication for future studies.

We present five critical discoveries for practitioners and risk communication researchers to consider.

### The power of narrative and narrative mechanisms

This study confirms that narratives are more powerful than nonnarrative risk messages at influencing the uptake of risk reduction behaviors (i.e. COVID-19 vaccination), when controlling for psychological and other factors (e.g. affective response, motivation to vaccinate, risk perceptions, COVID-19 experience, flu vaccine history, and demographics). In our study, all three narrative conditions had significant conditional effects on vaccine behavior in comparison to the control condition (Table 2, with indicator coding; Supplement A, with Helmert coding for the average of all narrative conditions compared with control). This finding underscores the need to craft risk messages in narrative form using characters, as opposed to simply positing a directive or moral.

Importantly, moving from this coarse scale to a finer one, how a narrative is structured is influential to decision making. In this study, the choice of victim—a narrative mechanism of character selection—matters. While previous work finds the hero character is particularly effective in persuading audiences (34), our study intentionally varies the victim character while the hero is held constant (i.e. the audience “protects” a victim—yourself, circle, or community—by getting vaccinated). While holding the hero constant across treatment narratives may have dampened the effect of the victim character selection, we nonetheless discover important differences in the impact of narratives with different victim characters. Risk messages focusing on the hero protecting their in-group (35) (i.e. “your circle”) are an effective strategy to influence vaccination behavior, suggesting the need for risk messaging aimed at motivating behavior by emphasizing the larger social or communal benefits of vaccination (Table 1).

### Nonlinear influence of narrative risk messages

The pathway of influence from risk message to behavior is not linear. Indeed, risk communication studies are inconsistent in finding direct effects on behavior (35), resulting in the need to develop more accurate explanatory models. The works of Slovic (36), Fischhoff (37), and others (38) have advanced risk communication models to include psychological factors in risk behavior decision making. The affect heuristic (39) is a well-established concept to predict risk perception and decisions, while the Theory of Planned Behavior (40) adds intention to act as an immediate antecedent of actual behavior. In this study, we build on these ideas by testing risk message effects in a serial mediation model. In other words, we assume the critical factors of affective responses and intended behavior do not operate in silos; instead, they mediate in a temporally important manner. Specifically, we find the influence of narrative-based messages on COVID-19 vaccine behavior to be serially mediated through affective response to the message and then motivation to vaccinate (i.e. intended behavior). In short, a risk message that generates a positive affective response results in higher motivation or intention to vaccinate, which then leads to higher COVID-19 vaccination behavior 8 weeks later. Thus, to be effective at influencing behavior, risk messages must first trigger the audience's attention, captured through a heightened affective response, which then activates an intention to act.

### The role of beliefs

Most decision models, including the Health Belief Model (41), account for the influence of a priori beliefs in decision making. Beliefs are informed by a variety of theories and measured in multiple ways, such as cultural beliefs (42); the beliefs, events, and values inventory (BEVI) (43); and political ideology (44). In the context of COVID-19, we chose to examine political ideology as the moderating belief system, given the increasingly politicized nature of science (45) and vaccines (46) in the US context. Political ideology is conceived of as a stable set of beliefs centered on the role of government in setting policies. Despite some criticism (47), political ideology is often operationalized on a conservative to liberal scale, with the former embracing limited government and the latter favoring government's role in public well-being and equity. With vaccine hesitancy known to be more associated with conservative ideology (48), we tested for the moderation of these beliefs on COVID-19 vaccine behavior. In our study, political ideology did not have a pervasive moderating effect. One potential explanation may be due to the timing of our data collection. Our panel survey was in the field mid-January—early February 2021 for *T*_1_ and mid-March—early April 2021 for *T*_2_. These were the first months following the Emergency Use Authorization of the COVID-19 vaccine on December 11, 2020, and individual perceptions and intentions were still forming.

However, there was a moderating effect for conservatives that merits our attention. When examining the moderation of total effects of treatment messages on vaccine behavior (Fig. 2), conservative respondents exposed to the “protect yourself” message had lower vaccine rates in comparison to both the control condition and liberal leaning respondents (Table 1 and Supplement C). We suspect this result among conservatives is due to a combination of risk perception and aversion to paternalism. In our study, conservative participants consider themselves less susceptible to the risks of COVID-19 than liberals (Supplement C) and thus may have rejected the message to get the vaccine to protect themselves, since their perception of the risk is low. On a theoretical level, the values of personal responsibility and autonomy are important touchstones for contemporary conservatives (49), thereby engendering a resistance to being told what to do.

Perhaps more importantly, conservative respondents had a higher level of vaccination with messages focused on protecting others (the average of “protect your circle” and “protect your community” messages) than the “protect yourself” message. This finding is surprising, given that prior literature routinely finds that conservatives are more individualistic (50, 51). Whereas previous studies have documented widespread vaccine hesitancy among conservatives (52) and questioned whether interventions such as outreach campaigns are enough to significantly move the needle toward greater vaccine uptake, our finding suggests a potential path forward for public health officials. Specifically, this study underscores the potential for positive effects across political ideologies of messages focusing on the communal benefit of vaccinations as opposed to strictly emphasizing individual concerns.

### The two dimensions of risk perception

Most risk communication studies suggest risk perceptions are a critical precursor to risk reduction behaviors (53). They often measure the extent to which messages influence risk perceptions, but there is conflicting evidence as to the influence of risk perceptions on risk reduction behaviors (54). We assess risk perceptions of COVID-19 (the risk agent), as opposed to risk perceptions of the vaccine itself (moral/solution). As such, in this study, risk perception is positioned as a covariate, as opposed to a mediating variable. Importantly, we partition risk perception into its two dimensions—likelihood and severity—as separate variables and find they operate differently. The perception of the severity of the impacts of COVID-19 influences greater affective responses, whereas the likelihood of getting COVID-19 impacts motivation to get the vaccine. Neither of the dimensions directly influences behavior. This result has important implications, as the two dimensions of risk perception operate independently along different conditional pathways as opposed to in a harmonious fashion.

### Visual policy narratives

Finally, risk communication must join the age of visually based media as an efficient and effective avenue for information dissemination and sharing. This requires risk messages to be visual *and* substantive. Our use of visual policy narrative messages has demonstrated that narratives can indeed influence risk reduction behavior in comparison to a nonnarrative visual control in the context of a graphic platform of communication. There is a robust need for future work in discovering not just what message structures are impactful, but what venues of dissemination create the amplification desired (55).

In sum, stories are an integral part of the human experience. They shape how we perceive the world around us, make sense of our history, and, ultimately, render decisions that affect ourselves and others. The advent of social media and other forms of visual-based communication have only magnified the importance of storytelling through new and improved pathways for disseminating information that is often embedded within complex and multifaceted visual environments. Responding to the call from the public health community, we find that visual narrative risk communication is an effective approach to encourage risk mitigation behavior. Attending to specific narrative mechanisms that enhance narrative power, as well as to the complex pathways through which narratives differentially impact their audiences, provides new insights that improve public health communication and behavior across the political spectrum.

## Materials and methods

Our research team conducted a three-wave online panel survey through Qualtrics^XM^ of individuals in 50 US states and Washington D.C. in 2021. We contracted with Qualtrics^XM^ to administer the survey using their proprietary panels of online survey respondents. These panel participants are recruited from a variety of sources, including website intercept recruitment, targeted email lists, gaming sites, customer loyalty web portals, and social media. In light of the well-documented and persistent disparities in both COVID-19's disease burden (56) and vaccine uptake (57), we intentionally oversampled individuals who identified as Hispanic (33.5%) and non-Hispanic Black (28.6%), compared to non-Hispanic White respondents (24.4%). Our novel sampling scheme helps ensure our findings are generalizable to those communities disproportionately impacted by the disease. Moreover, although our sample is thus not statistically representative of the US adult population, the experimental results of our study are internally valid given our randomized study design and control for demographic variables (see Supplement D for randomization check).

For each wave, the informed consent started with the purpose of the survey, which was to understand attitudes, perceptions, beliefs, experiences, and behaviors about COVID-19. Here, we present data from *T*_1_ (*n* = 3,900; launched between January 11 and February 3, 2021) and *T*_2_ (*n* = 2,268; launched between March 22 and April 9, 2021). In these survey waves, we conducted an experiment wherein respondents were randomly assigned to one of four experimental conditions in *T*_1_, comprised of three visual policy narrative treatment conditions (“protect yourself,” *n* = 986, 25%; “protect your circle,” *n* = 974, 25%; and “protect your community,” *n* = 955, 25%) and a control condition (“get the vaccine,” *n* = 969, 25%) (Fig. 4).

*Message* condition at *T*_1_ is the predictor variable (*X_i_*) (Figs. 2 and 3). About 2 months later in *T*_2_, we then measured *vaccine behavior* (*Y*_1_) as the outcome variable, with respondents choosing yes or no to having received one or more COVID-19 vaccine doses. We measured two potential mediating variables immediately after respondents were exposed to the message conditions during *T*_1_. The first assessed respondents’ *affective reaction* (*M*_1_) to the visual message, on a 7-point scale from “extremely negative” to “extremely positive”; the second measured the extent to which the respondent felt the message *motivated them to vaccinate* (*M*_2_), on a 7-point scale from “not at all” to “a great deal.” *Political ideology* serves as a potential moderator (*W*), with lower numbers associated with stronger conservative beliefs and higher numbers associated with stronger liberal beliefs on a 7-point scale. All covariates were measured in *T*_1_, before exposure to experimental conditions. Along with demographics, covariates include the two dimensions of risk perception: likelihood and severity. Importantly, risk perception was not used as a mediating variable in the model, as the risk perception surrounded the impacts of getting the coronavirus, not the COVID-19 vaccine. These risk perception variables are multi-indicator variables, each having robust reliability with Cronbach's alphas greater than 0.80. Given the importance of experience in risk decisions, respondents’ COVID-19 experience and the frequency with which they get the seasonal flu vaccine are included. Table 4 provides a summary of all variables.

We conduct two main analyses. Analysis 1 assessed the overall effect of narrative risk messages (*X_i_*) on vaccination (*Y*_1_), moderated by political ideology (*W*) (Fig. 2), controlling for covariates risk perception, COVID-19 experience, flu vaccine behavior, and demographics. In other words:

- moderation of political ideology (*W*) on the message (*X_i_*) → vaccine behavior (*Y*_1_), statistically defined as the coefficient of the *X* * *W* interaction term in the regression predicting *Y*_1_ when the mediators *M*_1_ and *M*_2_ are also included in the model.

Analysis 2 was a moderated serial mediation analysis with conditional effects (Fig. 2). In Analysis 2, we test (i) the mediation effects of message conditions (*X_i_*) through affective response (*M*_1_) and motivation to vaccinate (*M*_2_) on vaccine behavior at *T*_2_ (*Y*_1_), both as individual mediators and as serial mediators, and (ii) the moderation effects of political beliefs (*W*) for these mediation effects, as well as for the direct effect of message conditions on affective response (*M*_1_), motivation to vaccinate (*M*_2_), and on vaccine behavior (*Y*_1_), all controlling for covariates risk perception, COVID-19 experience, flu vaccine behavior, and demographics (Fig. 2). In other words, Analysis 2 tests three mediational pathways, each moderated by political ideology:

- message (*X_i_*) → affective response (*M*_1_) → vaccine behavior (*Y*_1_), statistically defined as the product of coefficients *a*_1_ * *b*_1_;
- message (*X_i_*) → motive to vaccinate (*M*_2_) → vaccine behavior (*Y*_1_), statistically defined as the product of coefficients *a*_2_ * *b*_2_;
- message (*X_i_*) → affective response (*M*_1_) → motive to vaccinate (*M*_2_) → vaccine behavior (*Y*_1_), statistically defined as the product of coefficients *a*_1_ * *d*_21_ * *b*_2_.

For the analysis, we use Hayes’ PROCESS models (30), a regression-based moderated mediation model, the regression coefficients of which are estimated using OLS regression (when *M*_1_ or *M*_2_ are the outcome variables) and logit regression analysis (when *Y*_1_ is the dichotomous outcome variable). For all models, we use 95% confidence intervals, and 5,000 bootstrap samples to generate bias-corrected confidence intervals. Given that the *X_i_* variable is a multicategorical variable, both Analysis 1 used two kinds of coding to represent the experimental conditions. The first is indicator coding (p. 70), which compares each treatment condition to the control.

*X*_1_ = protect yourself compared to control
*X*_2_ = protect the circle compared to control
*X*_3_ = protect the community compared to control

The second is Helmert contrast coding (p. 226), which is a successive comparison of conditions.

*X*_4_ = the average of all three narrative message conditions compared to control
*X*_5_ = “protect the circle” plus “protect the community” vs. “protect yourself”
*X*_6_ = “protect the community” compared to “protect the circle”

Thus, in the full statistical model, each path from *X_i_* to other variables illustrated in Figs. 2 and 3 includes three different paths, each representing the path from *X_i_* to other variables. Similarly, each moderating effect of *W* includes interaction terms with *X_i_*.

## Supplementary Material

pgad080_Supplementary_Data

## Data Availability

Data citation: Shanahan, Elizabeth et al. (2023), Visual policy narrative messaging and COVID-19 vaccine uptake, Dryad, Dataset, https://doi.org/10.5061/dryad.fn2z34v0c.
