# Correction to: Visual policy narrative messaging improves COVID-19 vaccine uptake

**DOI:** 10.1093/pnasnexus/pgad273

**Published:** 2023-08-22

This is a correction to: Elizabeth A Shanahan and others, Visual policy narrative messaging improves COVID-19 vaccine uptake, PNAS Nexus, Volume 2, Issue 4, April 2023, pgad080, https://doi.org/10.1093/pnasnexus/pgad080

After publication it was discovered that this paper was missing a statement acknowledging compliance with the PNAS Nexus Human and Animal Participants and Clinical Trials policy:

Our research team conducted a three-wave online panel survey through QualtricsXM of individuals in 50 US states and Washington D.C. in 2021. We contracted with QualtricsXM to administer the survey using their proprietary panels of online survey respondents. This humans subjects research was approved by Bentley University as exempt under Protection of Human Research Subjects, 45 CFR Part 46.104 [d] (2) (i); the certification is IRB #10202024. Informed consent was obtained by each participant. These panel participants are recruited from a variety of sources, including website intercept recruitment, targeted email lists, gaming sites, customer loyalty web portals, and social media.

