# Correction to: Visual policy narrative messaging improves COVID-19 vaccine uptake

**DOI:** 10.1093/pnasnexus/pgag199

**Published:** 2026-06-19

This is a correction to: Elizabeth A Shanahan, Rob A DeLeo, Elizabeth A Albright, Meng Li, Elizabeth A Koebele, Kristin Taylor, Deserai Anderson, Katherine L Dickinson, Honey Minkowitz, Thomas A Birkland, Manli Zhang, Visual policy narrative messaging improves COVID-19 vaccine uptake, *PNAS Nexus*, Volume 2, Issue 4, April 2023, pgad080, https://doi.org/10.1093/pnasnexus/pgad080

A change has been made in the authorship list from that originally published.

