# Supplementary material for: Visual policy narrative messaging improves COVID-19 vaccine uptake
Source: PNAS Nexus. 2023 Apr 18;2(4):pgad080. doi: 10.1093/pnasnexus/pgad080 (PMC10122412; doi:10.1093/pnasnexus/pgad080)
Supplement: pgad080_Supplementary_Data [file pgad080_supplementary_data.zip › PNASNEXUS-PNASNEXUS-2022-00667-s02.docx]

Visual policy narrative messaging improves COVID-19 vaccine uptake

Elizabeth A. Shanahan*, Montana State University, U.S. [shanahan@montana.edu](mailto:shanahan@montana.edu)

Rob A. DeLeo, Bentley University, U.S. [rdeleo@bentley.edu](mailto:rdeleo@bentley.edu)

Elizabeth Albright, Duke University, U.S. [elizabeth.albright@duke.edu](mailto:elizabeth.albright@duke.edu)

Meng Li, University of Colorado—Denver, U.S. [meng.li@ucdenver.edu](mailto:meng.li@ucdenver.edu)

Elizabeth A. Koebele, University of Nevada—Reno, U.S. [ekoebele@unr.edu](mailto:ekoebele@unr.edu)

Kristin Taylor, Wayne State University, U.S. [kristin.taylor@wayne.edu](mailto:kristin.taylor@wayne.edu)

Deserai Crow, University of Colorado—Denver, U.S. [deserai.crow@ucdenver.edu](mailto:deserai.crow@ucdenver.edu)

Katherine L. Dickinson, University of Colorado—Denver, U.S. [katherine.dickinson@cuanschutz.edu](mailto:katherine.dickinson@cuanschutz.edu)

Honey Minkowitz, North Carolina State University, U.S. [hminkow@ncsu.edu](mailto:hminkow@ncsu.edu)

Tom Birkland, North Carolina State University, U.S. [tabirkla@ncsu.edu](mailto:tabirkla@ncsu.edu)

Manli Zhang, University of Colorado—Denver, U.S. [manli.zhang@ucdenver.edu](mailto:manli.zhang@ucdenver.edu)

*Corresponding author

**Supplement A**

| \| **SA1. Helmert Coding Moderated Mediation Models with Interactions and Covariates** \| \| \| \| \| --- \| --- \| --- \| --- \| \| **Variable** \| **Model 1**  Affective response (M_1_;T_1_)  *b* (se) \| **Model 2**  Motivated to vaccinate (M_2_;T_1_)  *b* (se) \| **Model 3**  Got COVID-19 vaccine (Y_1_;T_2_)  *b* (se) \| \| Constant \| 3.176*** (.1835) \| -1.0338*** (.2046) \| -5.1131*** (.3767) \| \| X_4_ \| .6814*** (.0794) \| -.1726* (.0838) \| -.0948 (.1400) \| \| X_5_ \| .0790 (.0822) \| .1106 (.0851) \| .2792 (.1425) \| \| X_6_ \| .0585 (.0957) \| -.0310 (.0990) \| -.2055 (.1636) \| \| Affective response (M_1_) \|  \| .8154*** (.0241) \| .0145 (.0523) \| \| Motivated to vaccinate (M_2_) \|  \|  \| .1701*** (.0390) \| \| X_4_ * political ideology \| -.0574 (.0505) \| .0085 (.0522) \| .1000 (.0869) \| \| X_5_* political ideology \| .0833 (.0527) \| .0244 (.0545) \| -.1685 (.0916) \| \| X_6_* political ideology \| -.0255 (.0607) \| .0499 (.0628) \| .0100 (.1042) \| \| Risk perception: likelihood \| .0049 (.0110) \| .0311** (.0114) \| .0026 (.0187) \| \| Risk perception: severity \| .0575*** (.0089) \| .0180 (.0093) \| .0243 (.0158) \| \| COVID-19 experience \| .1190 (.1021) \| -.1744 (.1057) \| .1544 (.1726) \| \| Flu vaccine frequency \| .2193*** (.0293) \| .1925*** (.0308) \| .4320*** (.0493) \| \| Race \| -.1862* (.0928) \| -.1149 (.0960) \| .4140** (.1566) \| \| Gender \| -.1876* (.0742) \| -.2059** (.0768) \| .0195 (.1270) \| \| Age \| .0119*** (.0025) \| .0069** (.0026) \| .0442*** (.0043) \| \| Education \| .0289 (.0266) \| .0120 (.0275) \| .1580*** (.0456) \| \| Income \| .0217 (.0145) \| -.0100 (.0150) \| .0736** (.0248) \| \| Kids \| -.0540 (.0366) \| .0511 (.0379) \| -.3167*** (.0696) \| \| Political ideology \| .0842*** (.0225) \| -.0047 (.0234) \| .0368 (.0391) \| | | | | | |
| --- | --- | --- | --- | --- | --- | --- | --- | --- | --- | --- | --- | --- | --- | --- | --- | --- | --- | --- | --- | --- | --- | --- | --- | --- | --- | --- | --- | --- | --- | --- | --- | --- | --- | --- | --- | --- | --- | --- | --- | --- | --- | --- | --- | --- | --- | --- | --- | --- | --- | --- | --- | --- | --- | --- | --- | --- | --- | --- | --- | --- | --- | --- | --- | --- | --- | --- | --- | --- | --- | --- | --- | --- | --- | --- | --- | --- | --- | --- | --- | --- | --- | --- | --- | --- | --- | --- | --- | --- | --- | --- | --- | --- | --- |
| **SA2. Helmert Coding Indirect Effects and Indices of Moderated Mediation** | | | | | |
| message🡪 affective response🡪 received a COVID-19 vaccine | | | | | |
| Indicator | Political ideology | Effect | BootSE | BootLLCI | BootULCI |
| X_4_ | -1.57 | .0112 | .0418 | -.0716 | .0928 |
| X_4_ | 0.00 | .0099 | .0369 | -.0613 | .0827 |
| X_4_ | +1.57 | .0086 | .0328 | -.0556 | .0759 |
| X_5_ | -1.57 | -.0008 | .0070 | -.0173 | .0124 |
| X_5_ | 0.00 | .0011 | .0059 | -.0106 | .0149 |
| X_5_ | +1.57 | .0030 | .0127 | -.0207 | .0317 |
| X_6_ | -1.57 | .0014 | .0092 | -.0181 | .0217 |
| X_6_ | 0.00 | .0008 | .0059 | -.0111 | .0142 |
| X_6_ | +1.57 | .0003 | .0071 | -.0143 | .0167 |
| Indicator | Index of moderated mediation | | BootSE | BootLLCI | BootULCI |
| X_4_ | -.0008 | | .0045 | -.0113 | .0081 |
| X_5_ | .0012 | | .0053 | -.0088 | .0136 |
| X_6_ | -.0004 | | .0036 | -.0086 | .0074 |
| message🡪 motivated to vaccinate🡪 received a COVID-19 vaccine | | | | | |
| Indicator | Political ideology | Effect | BootSE | BootLLCI | BootULCI |
| X_4_ | -1.57 | -.0316 | .0211 | -.0767 | .0059 |
| **X_4_** | **0.00** | **-.0293** | **.0159** | **-.0646** | **-.0021** |
| X_4_ | +1.57 | -.0271 | .0216 | -.0747 | .0106 |
| X_5_ | -1.57 | .0123 | .0216 | -.0278 | .0575 |
| X_5_ | 0.00 | .0188 | .0160 | -.0100 | .0535 |
| X_5_ | +1.57 | .0253 | .0237 | -.0168 | .0783 |
| X_6_ | -1.57 | -.0186 | .0256 | -.0736 | .0276 |
| X_6_ | 0.00 | -.0053 | .0176 | -.0423 | .0281 |
| X_6_ | +1.57 | .0081 | .0263 | -.0442 | .0599 |
| Indicator | Index of moderated mediation | | BootSE | BootLLCI | BootULCI |
| X_4_ | .0014 | | .0091 | -.0171 | .0193 |
| X_5_ | .0042 | | .0102 | -.0159 | .0252 |
| X_6_ | .0085 | | .0121 | -.0143 | .0342 |
| message🡪 affective response🡪 motivated to vaccinate🡪 received a COVID-19 vaccine | | | | | |
| Indicator | Political ideology | Effect | BootSE | BootLLCI | BootULCI |
| **X_4_** | **-1.57** | **.1070** | **.0310** | **.0543** | **.1751** |
| **X_4_** | **0.00** | **.0945** | **.0255** | **.0509** | **.1501** |
| **X_4_** | **+1.57** | **.0820** | **.0271** | **.0376** | **.1443** |
| X_5_ | -1.57 | -.0072 | .0167 | -.0419 | .0260 |
| X_5_ | 0.00 | .0109 | .0114 | -.0102 | .0355 |
| X_5_ | +1.57 | .0291 | .0167 | -.0003 | .0662 |
| X_6_ | -1.57 | .0137 | .0205 | -.0252 | .0563 |
| X_6_ | 0.00 | .0081 | .0131 | -.0167 | .0362 |
| X_6_ | +1.57 | .0025 | .0181 | -.0333 | .0391 |
| Indicator | Index of moderated mediation | | BootSE | BootLLCI | BootULCI |
| X_4_ | -.0080 | | .0089 | -.0274 | .0092 |
| X_5_ | .0116 | | .0077 | -.0024 | .0283 |
| X_6_ | -.0035 | | .0091 | -.0228 | .0143 |

Note: * *p*<.05; ** *p*< .01; *** *p*<.001

X_4_ *narrative* (average of *protect yourself*, *protect your circle*, and *protect your community*) compared to *control*

X_5_ average of *protect circle* and *protect your community* compared to *protect yourself*

X_6_ *protect community* compared to *protect your circle*

Level of confidence for all confidence intervals = 95

Number of bootstrap samples for percentile bootstrap confidence intervals: 5000

Political ideology was mean centered

-1.57 = 1SD below the mean on political ideology

0.00 = the mean on political ideology

+1.57 = 1 SD above the mean on political ideology

Level of confidence for all confidence intervals = 95

Number of bootstrap samples for percentile bootstrap confidence intervals: 5000

Hayes (2022) Model 85

**Supplement B**

| **SB1. Indicator Coding Indirect Effects and Indices of Moderated Mediation** | | | | | |
| --- | --- | --- | --- | --- | --- |
| message🡪 affective response🡪 received a COVID-19 vaccine | | | | | |
| Indicator | Political ideology | Effect | BootSE | BootLLCI | BootULCI |
| X_1_ | -1.57 | .0117 | .0449 | -.0765 | .1039 |
| X_1_ | 0.00 | .0091 | .0347 | -.0603 | .0778 |
| X_1_ | +1.57 | .0065 | .0258 | -.0457 | .0622 |
| X_2_ | -1.57 | .0102 | .0398 | -.0675 | .0941 |
| X_2_ | 0.00 | .0098 | .0376 | -.0644 | .0843 |
| X_2_ | +1.57 | .0094 | .0364 | -.0635 | .0826 |
| X_3_ | -1.57 | .0116 | .0447 | -.0765 | .1034 |
| X_3_ | 0.00 | .0107 | .0406 | -.0698 | .0922 |
| X_3_ | +1.57 | .0097 | .0374 | -.0639 | .0868 |
| Indicator | Index of moderated mediation | | BootSE | BootLLCI | BootULCI |
| X_1_ | -.0016 | | .0074 | -.0188 | .0125 |
| X_2_ | -.0002 | | .0042 | -.0104 | .0080 |
| X_3_ | -.0006 | | .0046 | -.0112 | .0087 |
| message🡪 motivated to vaccinate🡪 received a COVID-19 vaccine | | | | | |
| Indicator | Political ideology | Effect | BootSE | BootLLCI | BootULCI |
| X_1_ | -1.57 | -.0398 | .0270 | -.0983 | .0083 |
| **X_1_** | **0.00** | **-.0419** | **.0203** | **-.0871** | **-.0071** |
| X_1_ | +1.57 | -.0440 | .0275 | -.1042 | .0029 |
| X_2_ | -1.57 | -.0182 | .0253 | -.0693 | .0328 |
| X_2_ | 0.00 | -.0204 | .0187 | -.0590 | .0153 |
| X_2_ | +1.57 | -.0227 | .0276 | -.0818 | .0282 |
| X_3_ | -1.57 | -.0368 | .0262 | -.0927 | .0099 |
| X_3_ | 0.00 | -.0257 | .0185 | -.0660 | .0076 |
| X_3_ | +1.57 | -.0146 | .0250 | -.0673 | .0321 |
| Indicator | Index of moderated mediation | | BootSE | BootLLCI | BootULCI |
| X_1_ | -.0013 | | .0115 | -.0251 | .0210 |
| X_2_ | -.0014 | | .0119 | -.0261 | .0206 |
| X_3_ | .0071 | | .0112 | -.0144 | .0299 |
| message🡪 affective response🡪 motivated to vaccinate🡪 received a COVID-19 vaccine | | | | | |
| Indicator | Political ideology | Effect | BootSE | BootLLCI | BootULCI |
| **X_1_** | **-1.57** | **.1118** | **.0340** | **.0541** | **.1891** |
| **X_1_** | **0.00** | **.0872** | **.0254** | **.0433** | **.1425** |
| **X_1_** | **+1.57** | **.0626** | **.0263** | **.0191** | **.1217** |
| **X_2_** | **-1.57** | **.0978** | **.0311** | **.0454** | **.1679** |
| **X_2_** | **0.00** | **.0941** | **.0262** | **.0481** | **.1508** |
| **X_2_** | **+1.57** | **.0904** | **.0302** | **.0386** | **.1583** |
| **X_3_** | **-1.57** | **.1115** | **.0349** | **.0522** | **.1886** |
| **X_3_** | **0.00** | **.1022** | **.0283** | **.0518** | **.1641** |
| **X_3_** | **+1.57** | **.0929** | **.0304** | **.0409** | **.1585** |
| Indicator | Index of moderated mediation | | BootSE | BootLLCI | BootULCI |
| X_1_ | -.0157 | | .0107 | -.0388 | .0033 |
| X_2_ | -.0023 | | .0101 | -.0223 | .0179 |
| X_3_ | -.0059 | | .0104 | -.0277 | .0140 |

X_1_ *protect yourself* compared to *control*

X_2_ *protect circle* compared to *control*

X_3_ *protect community* compared to *control*

Political ideology was mean centered

-1.57 = 1SD below the mean on political ideology

0.00 = the mean on political ideology

+1.57 = 1 SD above the mean on political ideology

Level of confidence for all confidence intervals = 95

Number of bootstrap samples for percentile bootstrap confidence intervals: 5000

**Supplement C**

**SC1. Mean Comparison of Risk Perception by Political Ideology**

| Risk perception | Political ideology | | Mean difference (se) | Lower bound | Upper bound |
| --- | --- | --- | --- | --- | --- |
| severity |  |  |  |  |  |
|  | conservative |  |  |  |  |
|  |  | moderate | -1.111*** (.166) | -1.51 | -.71 |
|  |  | liberal | -1.979*** (.174) | -2.40 | -1.56 |
| F = 64.823, *p* <.001, *df* = 2 | | | | | |
| likelihood |  |  |  |  |  |
|  | conservative |  |  |  |  |
|  |  | moderate | -.030 (.145) | -.38 | .32 |
|  |  | liberal | -.513** (.152) | -.88 | -.15 |
| F = 7.492, *p* <.001, *df* = 2 | | | | | |

Note: * *p*<.05; ** *p*< .01; *** *p*<.001

Political ideology was reduced from a seven-point scale to a three point scale, combining all conservative and liberal responses and maintaining the moderate responses.

**Supplement D**

**SD1. Randomization check**

| **GENDER** | | | | | |
| --- | --- | --- | --- | --- | --- |
|  | | | male | female non-binary | Total |
| Experimental condition | Control | Count | 475 | 491 | 966 |
|  |  | % within experimental condition | 49.2% | 50.8% | 100.0% |
|  |  | % of Total | 12.3% | 12.7% | 25.0% |
|  | Protect yourself | Count | 497 | 486 | 983 |
|  |  | % within experimental condition | 50.6% | 49.4% | 100.0% |
|  |  | % of Total | 12.8% | 12.6% | 25.4% |
|  | Protect your circle | Count | 470 | 502 | 972 |
|  |  | % within experimental condition | 48.4% | 51.6% | 100.0% |
|  |  | % of Total | 12.1% | 13.0% | 25.1% |
|  | Protect your community | Count | 478 | 472 | 950 |
|  |  | % within experimental condition | 50.3% | 49.7% | 100.0% |
|  |  | % of Total | 12.3% | 12.2% | 24.5% |
| Total | | Count | 1920 | 1951 | 3871 |
|  |  | % within experimental condition | 49.6% | 50.4% | 100.0% |
|  |  | % of Total | 49.6% | 50.4% | 100.0% |

| **Chi-Square Tests** | | | | | | | |
| --- | --- | --- | --- | --- | --- | --- | --- |
|  | | Value | | df | | Asymptotic Significance (2-sided) | |
| Pearson Chi-Square | | 1.231^a^ | | 3 | | .746 | |
| Likelihood Ratio | | 1.231 | | 3 | | .745 | |
| Linear-by-Linear Association | | .026 | | 1 | | .871 | |
| N of Valid Cases | | 3871 | |  | |  | |
| a. 0 cells (0.0%) have expected count less than 5. The minimum expected count is 471.20. | | | | | | | |
| **Symmetric Measures** | | | | | | |  |
|  | | | Value | | Approximate Significance | |  |
| Nominal by Nominal | Phi | | .018 | | .746 | |  |
|  | Cramer's V | | .018 | | .746 | |  |
| N of Valid Cases | | | 3871 | |  | |  |

| **RACE** | | | | | |
| --- | --- | --- | --- | --- | --- |
|  | | | All Other Races | White | Total |
| Experimental condition | Control | Count | 730 | 239 | 969 |
|  |  | % within experimental condition | 75.3% | 24.7% | 100.0% |
|  |  | % of Total | 18.8% | 6.2% | 24.9% |
|  | Protect yourself | Count | 743 | 243 | 986 |
|  |  | % within experimental condition | 75.4% | 24.6% | 100.0% |
|  |  | % of Total | 19.1% | 6.3% | 25.4% |
|  | Protect your circle | Count | 743 | 231 | 974 |
|  |  | % within experimental condition | 76.3% | 23.7% | 100.0% |
|  |  | % of Total | 19.1% | 5.9% | 25.1% |
|  | Protect your community | Count | 721 | 234 | 955 |
|  |  | % within experimental condition | 75.5% | 24.5% | 100.0% |
|  |  | % of Total | 18.6% | 6.0% | 24.6% |
| Total | | Count | 2937 | 947 | 3884 |
|  |  | % within experimental condition | 75.6% | 24.4% | 100.0% |
|  |  | % of Total | 75.6% | 24.4% | 100.0% |

| **Chi-Square Tests** | | | |
| --- | --- | --- | --- |
|  | Value | df | Asymptotic Significance (2-sided) |
| Pearson Chi-Square | .320^a^ | 3 | .956 |
| Likelihood Ratio | .322 | 3 | .956 |
| Linear-by-Linear Association | .054 | 1 | .817 |
| N of Valid Cases | 3884 |  |  |
| a. 0 cells (0.0%) have expected count less than 5. The minimum expected count is 232.85. | | | |

| **Symmetric Measures** | | | |
| --- | --- | --- | --- |
|  | | Value | Approximate Significance |
| Nominal by Nominal | Phi | .009 | .956 |
|  | Cramer's V | .009 | .956 |
| N of Valid Cases | | 3884 |  |

| **ANOVA. Age x Experimental Condition** | | | | | | |
| --- | --- | --- | --- | --- | --- | --- |
|  | | Sum of Squares | df | Mean Square | F | Sig. |
| AGE | Between Groups | 459.822 | 3 | 153.274 | .496 | .685 |
|  | Within Groups | 1198723.155 | 3880 | 308.949 |  |  |
|  | Total | 1199182.977 | 3883 |  |  |  |

| **ANOVA. Education x Experimental Condition** | | | | | | |
| --- | --- | --- | --- | --- | --- | --- |
|  | | Sum of Squares | df | Mean Square | F | Sig. |
| EDUCATION | Between Groups | 7.626 | 3 | 2.542 | 1.149 | .328 |
|  | Within Groups | 8581.666 | 3880 | 2.212 |  |  |
|  | Total | 8589.292 | 3883 |  |  |  |

| **ANOVA. Income x Experimental Condition** | | | | | | |
| --- | --- | --- | --- | --- | --- | --- |
|  | | Sum of Squares | df | Mean Square | F | Sig. |
| INCOME | Between Groups | .330 | 3 | .110 | .014 | .998 |
|  | Within Groups | 30831.129 | 3880 | 7.946 |  |  |
|  | Total | 30831.459 | 3883 |  |  |  |
